# Detecting the Hormonal Pathways in Oilseed Rape behind Induced Systemic Resistance by *Trichoderma harzianum* TH12 to *Sclerotinia sclerotiorum*

**DOI:** 10.1371/journal.pone.0168850

**Published:** 2017-01-03

**Authors:** Jawadayn Talib Alkooranee, Tamarah Raad Aledan, Ali Kadhim Ali, Guangyuan Lu, Xuekun Zhang, Jiangsheng Wu, Chunhua Fu, Maoteng Li

**Affiliations:** 1 Department of Biotechnology, College of Life Science and Technology, Huazhong University of Science and Technology, Wuhan, China; 2 Plant Protection, College of Agriculture, University of Basrah, Basrah, Iraq; 3 Hubei Collaborative Innovation Center for the Characteristic Resources Exploitation of Dabie Mountains, Huanggang Normal University, Huanggang, China; 4 Oil Crops Research Institute, Chinese Academy of Agricultural Sciences, Wuhan, Hubei, China; 5 National Key Laboratory of Crop Genetic Improvement, Huazhong Agricultural University, Wuhan, China; Wuhan Botanical Garden, CHINA

## Abstract

Plants have the ability to resist pathogen attack after infection or treatment with biotic and abiotic elicitors. In oilseed rape plant *Brassica napus* AACC and in the artificially synthesized *Raphanus alboglabra* RRCC, the root-colonizing *Trichoderma harzianum* TH12 fungus triggers induced systemic resistance (ISR), and its culture filtrate (CF) triggers a systemic acquired resistance (SAR) response against infection by the *Sclerotinia sclerotiorum*. Salicylic acid (SA) and jasmonic acid/ethylene (JA/ET) are plant hormone signals that play important roles in the regulation of ISR and SAR. In this study, at six different time points (1, 2, 4, 6, 8 and 10 days post-infection [dpi]), six resistance genes were used as markers of signaling pathways: JA/ET signaling used *AOC3*, *PDF1*.*2* and *ERF2* genes, while *PR-1*, *TGA5* and *TGA6* genes were used as markers of SA signaling. The results of quantitative real-time polymerase chain reaction (qRT-PCR) showed that *AOC3*, *PDF1*.*2* and *ERF2* expression levels in infected leaves of AACC and RRCC increase at 1 and 2 dpi with *S*. *sclerotiorum* or inoculation with TH12. *PR-1*, *TGA5* and *TGA6* expression levels increased at 8 and 10 dpi in infected leaves. *PR-1*, *TGA5* and *TGA6* expression levels increased early in plants treated with CF in both of the healthy genotypes. Furthermore, induction of SA- and JA/ET-dependent defense decreased disease symptoms in infected leaves at different times. The results suggest that the RRCC genotype exhibits resistance to disease and that the ability of TH12 and its CF to induce systemic resistance in susceptible and resistant oilseed rape genotypes exists. In addition, the results indicate for the first time that in RRCC the SA signaling pathway is involved in resistance to necrotrophic pathogens.

## Introduction

*Sclerotinia sclerotiorum* (Lib) de Bary is a necrotrophic lifestyle fungal pathogen that can cause diseases (i.e., sclerotinia stem rot [SSR] in more than 400 host plants belonging to 75 families, including oilseed rape). SSR is reported from most of the oilseed-producing areas of the world, including China, India, Iran, USA, Brazil, Canada, Germany, Italy, Sweden, France, Finland, England and Denmark [1–4]. *Sclerotinia sclerotiorum* causes 0–20% of yield loss every year in *Brassica napus* crops and can reach up to 80% in severely infected fields in China [5].

Furthermore, disease management through the use of fungicides is also ineffective due to the negative effects of fungicides on human health and on the environment in addition to difficulty in timing the chemical control application with the release of ascospores [6]. Resistant genotypes and biotic elicitor applications offer the only economic and sustainable methods for effectively managing SSR disease. Resistance to *S*. *sclerotiorum* also was identified in some genotype artificially synthesized in China, namely *Raphanus alboglabra* (RRCC, 2n = 34) [2], achieved through hybridization between *Raphanus sativus* and *B*. *alboglabra* (different genotypes of Brassicaceae family) [7, 8], and partial resistance was identified in *B*. *napus* AACC and *Brassica juncea* genotypes [9, 10].

The biotic elicitor is a beneficial nonpathogenic microbes present in rhizosphere of plant roots, is play an important role in improving the growth and protection of plants, it has a different mechanisms of elicitation that could induce the systemic resistance in hosts against pathogens through producing some materials as enzymes, polysacharids, hormones, secondary metabolites. etc, that act as a internal stimuli to resistance inside hosts, which plays an critical role in activation of the resistance genes and change the state from turn off to turn on. The induced systemic resistance (ISR) and Systemic acquired resistance (SAR) are two forms of resistance in plants caused by prior infection or treatment by biotic or abiotic elicitors that emerged as an important mechanisms that lead to resistance against subsequent challenge by the parasites, pathogens and insect herbivores, the combination of ISR and SAR can promote the protection process in plants in viz., *Arabidopsis*, radish, cucumber, tobacco and carnation against various pathogens [11].

Plant growth-promoting fungi (PGPF) and plant growth-promoting rhizobacteria (PGPR) are groups of free-living rhizosphere fungi/bacteria that colonize root systems and exert beneficial effects on plant growth and yield. These groups stimulate plant immunity to a wide range of pathogens, a process known as induced systemic resistance (ISR) [2, 12, 13, 14].

*Trichoderma* spp. is soil-borne, versatile opportunistic plant symbiont that induces growth and resistance to a wide variety of phytopathogens [13, 15]. It has been reported that some *Trichoderma* spp., can stimulate ISR in crops via signaling molecules, such as jasmonic acid (JA), ethylene (ET), and salicylic acid (SA), to plant diseases caused by biotrophic or necrotrophic pathogens, such as *S*. *sclerotiorum*, *Erysiphe cruciferarum*, *Fusarium verticillioides* and *Botrytis cinerea* [2, 16–20].

The protection provided by isolates of *Trichoderma* spp. that stimulate ISR in hosts can be as effective as that provided by chemical materials or fungicides [21]. Moreover, cell-free culture filtrates (CF) of *Trichoderma* spp. enhance systemic acquired resistance (SAR) in plants by stimulating signaling molecules in oilseed rape against a variety of pathogens. *Trichoderma harzianum* TH12 and its CF were identified as providing the most significant growth inhibition of the phytopathogen *S*. *sclerotiorum*, which reached 100% among 30 *Trichoderma* species [2, 16]. *Trichoderma* spp. have the ability to produce a broad diversity of important secondary metabolites, especially those that play a role in bio-control mechanisms, such as the following secondary metabolites: pyrones, koninginins, trichothecenes, heptelidic acid, viridins, harzialactones, derivatives, ergosterol derivatives, peptaibols, anthraquinones, azaphilones, trichodermamides, butenolides, daucanes, acoranes, isocyano metabolites, butenolides, diketopiperazines, viridiofungins, trichodenones, cyclopentenone derivatives, cyclonerodiol derivatives, statins, setin-like metabolites, bisorbicillinoids, nitrogen heterocyclic compounds and others [22, 23].

Plants respond actively to pathogen attack after being treated with biotic elicitors, and plants also possess the capacity to resist after being infected with a pathogen through the deployment of several mechanisms associated with resistance or defense-response genes; the speed of these responses depends on the resistance or sensitivity of the genotype to pathogens. Oilseed–pathogen interactions in the *R*. *alboglabra* RRCC resistant genotype and in a *B*. *napus* AACC susceptible genotype, which have been studied for defense responses to pathogens, are regulated by hormonal signaling pathways, which are considered pathways to resistance genes in plants, including the JA/ET and SA pathways [16].

The plant hormones JA, ET and SA are central regulators the resistance system in plants. JA and ET are important players in the regulation of ISR and have been proven to be effective against typical necrotrophic fungi attackers that are sensitive to JA/ ET-dependent defenses [11], while SA pathway is important player in the regulation of SAR, SA pathway most effective against biotrophic pathogens that are sensitive to SA-dependent defenses [16].

In earlier studies, it was found that genes related to pathogenesis, such as *PR-1*, *PR-2*, *PR-3*, *PR-4* and *PR-5*, were involved in the defense of resistant plants to *S*. *sclerotiorum* infection, which acts directly against fungal pathogens. Pathogenesis-related proteins, including *PR-1*, β-1,3-glucanase (*PR-2*) and *PR-5*, are often used as markers for the SA-dependent SAR, whereas some genes, including plant defense 1.2 (*PDF1*.*2*), basic chitinase (*PR-3*) and pathogenesis-related 4 (*PR-4*) genes, are used as markers of the JA-dependent SAR [16, 24]. TGA transcription factor 5 (*TGA5*) and TGA transcription factor 6 (*TGA6*) genes have been used as markers of the SA pathway, whereas the allene oxide cyclase 3 (*AOC3*) and ethylene response factor 2 (*ERF2*) genes have been used as markers of the ET pathway in *B*. *napus* and *Brassica carinata* in response to *S*. *sclerotiorum* challenge [25]. In most cases, necrotrophic pathogens are used as elicitors of the JA/ET signaling pathway, whereas the SA signaling pathway is activated by local infection with biotrophic pathogens, and SA signaling causes SAR [25–28]. The JA/ET and SA signaling pathways have been proven to optimize the defense response against an attacker [29, 30]. In addition, the rhizosphere-competent, non-pathogenic organisms lead to the accumulation of JA/ET and cause resistance (i.e., ISR) [31].

In this study, we will examine the response speed of resistance genes (*PR-1*, *TGA5*, *TGA6*, *AOC3*, *PDF1*.*2* and *ERF2*) and JA/ET and SA signaling pathways in two different genotypes of oilseed rape (RRCC is resistant and AACC is sensitive to *S*. *sclerotiorum* infection) with and without treatment with TH12 and its CF during different periods of time– 1, 2, 4, 6, 8 and 10 days post infection (dpi).

## Materials and Methods

### Plant and fungal materials

*R*. *alboglabra* RRCC and *B*. *napus* AACC genotypes, and the fungal pathogen *S*. *sclerotiorum*, in addition to *T*. *harzianum* TH12 and its CF as a biotic elicitor, used in this study were stored in our research group laboratory [2, 16].

The laboratory and greenhouse experiments were conducted in the Biotechnology Department, School of Life Science and Technology, Huazhong University of Life Science and Technology, Wuhan, China. TH12 was collected from a soil samples located in rapeseed fields in Wuhan, Hubei province of China (114°25'39.7"E; 30°30'39.8"N) in 2014 as described in detail before [2]. *Sclerotinia sclerotiorum* and TH12 were grown on potato dextrose agar (200 g sliced potatoes, 20 g dextrose and 20 g agar powder in 1000 mL water). We used AACC and RRCC as oilseed rape-susceptible and oilseed rape-resistant genotypes, respectively; TH12 and CF as biotic elicitors; and *S*. *sclerotiorum* as a necrotrophic lifestyle fungal pathogen.

Field experiments were conducted in oilseed rape fields at Huazhong Agricultural University, when plants were flowering (90 days) during spring 2015.

#### Production of cell-free CF of *T*. *harzianum* TH12

Cell-free CF from 20-day-old TH12 grown on PDB at 25 ± 2°C, then were prepared by centrifugation (12,000 × g for 15 min), followed by filter sterilization with a 0.4 μm filter unit, and then the supernatants were collected. The method employed for the production of CF of *T*. *harzianum* TH12 is given in [16].

#### Plant cultivation under greenhouse and field conditions

Six seeds of AACC and RRCC were sown in a mixture of 1:1 clear sand and peat moss by volume, in 25-cm-diameter plastic pots that were sterilized twice for 30 min within a 24-h period under greenhouse conditions at 18/14 (±1)°C (day/night) temperature and a light intensity of 150 μE/m^2^/s for 12-h light/dark cycles. Seedlings were irrigated twice a week as described in detail previously [2].

#### Plant treatment with biotic elicitors and *S*. *sclerotiorum* infection

To screen TH12 and CF biotic elicitors capable of eliciting ISR by greenhouse experiments, 30-day-old AACC and RRCC genotypes were treated with 10-mL suspensions of *T*. *harzianum* TH12 (1.5 × 10^7^ CFU/mL) and its CF by blending and then mixing with the upper soil surface; other seedlings were treated with water as a control treatment. Mycelial 1-cm^2^ agar discs of 3-day-old *S*. *sclerotiorum* grown on potato dextrose agar were collected from an active colony edge. The infection of AACC and RRCC was determined by inoculating mycelial agar discs that had been collected previously and upended onto the adaxial surface of both genotypes on the first and second true leaves 1 day after being treated with the biotic elicitor. Leaves of control plants were treated similarly with 1-cm^2^ potato dextrose agar discs without mycelial growth. The inoculated genotype seedlings were covered with moistened foil after inoculation, the non-infected seedlings were transferred to a “clean” growth chamber and the infected seedlings were returned to the greenhouse. Each treatment consisted of three replicates.

Leaves of both genotypes were harvested from three plants per treatment at 1, 2, 4, 6, 8 and 10 dpi, with five seedlings per pot in three replicates (pots) for each time point. Agar plugs and pathogen tissues were then removed, and liquid nitrogen was used to freeze the leaves. RNA extraction and subsequent analyses were carried out on leaves stored at -80°C.

In the field, two inoculation procedures of leaves and stems were carried out on both genotypes (90 days old) as described in detail previously [5], with some minor modifications to assess the resistance to the pathogen. The lesion length along the leaves was measured at 1, 2, 4, 6, 8 and 10 dpi, and the lesion length along the stems of both genotypes was measured at 1, 2, 4, 6, 8, 10 and 30 dpi.

#### Gene expression assay

Isolation of total RNA, first-strand cDNA synthesis and quantitative real-time polymerase chain reaction (qRT-PCR) were used as described in detail previously [16]. Six primers (Table 1) were used for AACC and RRCC, including the *AOC3*, *PDF1*.*2*, *ERF2*, *PR-1*, *TGA5* and *TGA6* genes, in addition to the *GAPDH* gene, which was used as a housekeeping marker gene. The *AOC3*, *PDF1*.*2* and *ERF2* genes were used as markers for the JA, JA/ET and ET signaling pathways, respectively, whereas for the SA signaling pathway, we used the *PR-1*, *TGA5* and *TGA6* genes.

**Table 1:** PCR primers used in the present study.

	Gene description	Primer sequence (5ʹ-3ʹ)
***PR-1***	Pathogenesis-related protein 1	Forward: AAAGCTACGCCGACCGACTACGAG
		Reverse: CCAGAAAAGTCGGCGCTACTCCA
***TGA5***	TGA transcription factor5	Forward: CGACGTCTTATCGGAGATTGG
		Reverse: TGTTCCGTCAATGGTTCCAC
***TGA6***	TGA transcription factor 6	Forward: CAGCCAAGAATGATGTCTTCCA
		Reverse: CCCACCAAGCCACAAGAAAC
***AOC3***	Allene Oxide Cyclase 3	Forward: CAAACCAAGTTCCAAGTCTTCC
		Reverse: GTATTCCACCAACACAGCGTTA
***PDF1*.*2***	Plant Defensin 1.2	Forward: TCCATCATCACCCTTCTCTTCGC
		Reverse: CCATGTTGTGCTCCTTCAAGTCG
***ERF2***	Ethylene Response Factor 2	Forward: ATGTACGGACAGAGCGAGGT
		Reverse: AAGCTTCGAAACCAACAAGTAACTG
***GAPDH***	Glyceraldehyde 3-phosphate dehydrogenase	Forward: CGCTTCCTTCAACATCATTCCCA
		Reverse: TCAGATTCCTCCTTGATAGCCTT

#### Statistical analysis

The experimental design of the greenhouse and field experiments was completely randomized, with three replicates for all treatments. Data of the results were subjected to analysis of variance using GenStat software, and the means (P < 0.05) were compared between treatments of oilseed rape genotypes, TH12, CF treatments and SSR disease using least significant difference tests [32].

## Results

### Symptoms in AACC and RRCC upon *S*. *sclerotiorum* infection

**Fig 1:**
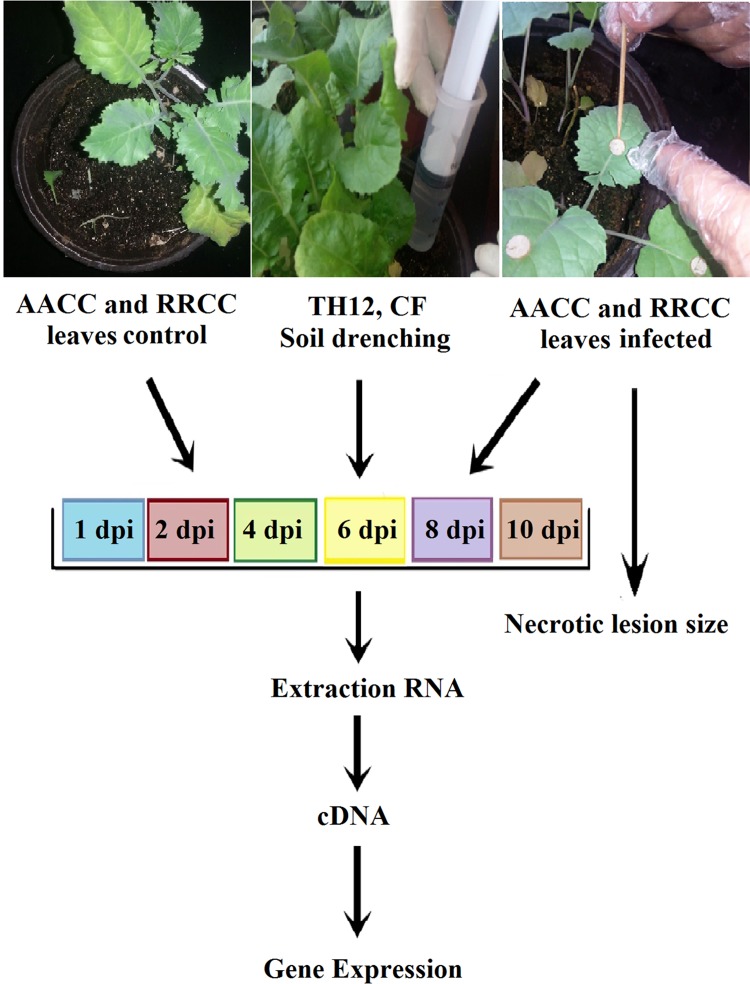
After 30 days of growth, pots of AACC and RRCC plants were treated with 10-mL suspensions of *T*. *harzianum* TH12 (1.5 ×10^7^ CFU/mL) or with its CF by blending with the upper soil surface, and other pots containing each genotype were not treated (served as controls). After 1 day, all leaves of the genotypes were infected with 1-cm^2^ mycelial agar discs of *S*. *sclerotiorum*. Leaves were collected at 1, 2, 4, 6, 8 and 10 dpi to measure the size of necrotic lesions or pooled for RNA extraction. Five seedlings per pot in three replicates (pots) for each time point and for each treatment were used in this study.

**Fig 2:**
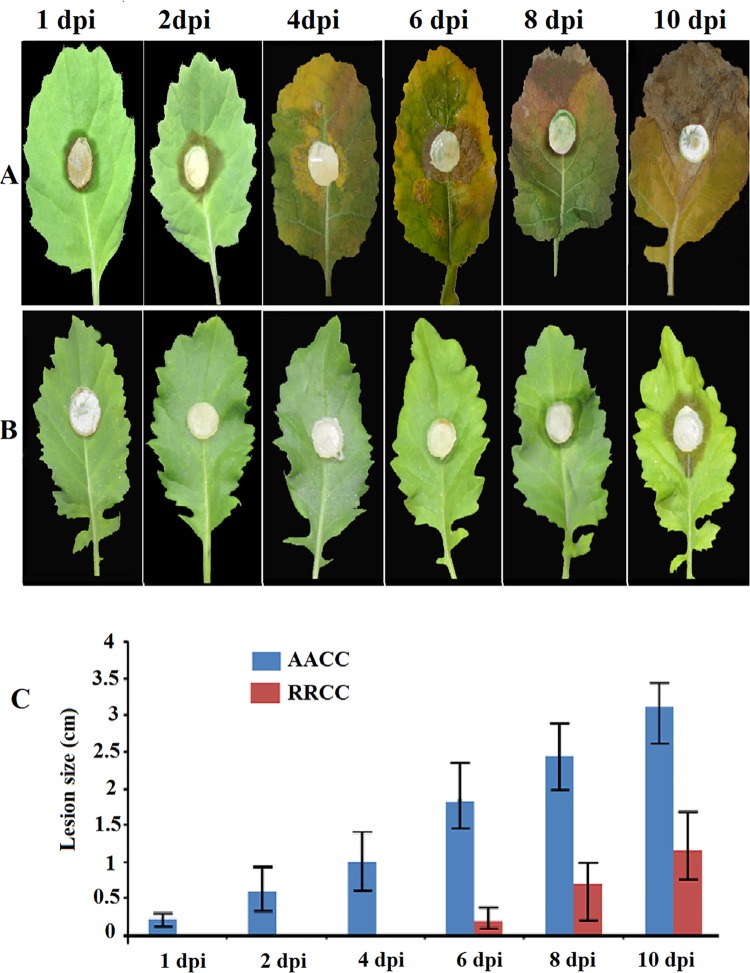
Disease progression of *S*. *sclerotiorum* infection on leaves of two genotypes: (A) *B*. *napus* AACC and (B) *R*. *alboglabra* RRCC. The leaves of both genotypes (30 days of growth) were infected with 1-cm^2^ mycelial agar discs of *S*. *sclerotiorum*. (C) Lesion size was measured 1, 2, 4, 6, 8 and 10 dpi.

*Sclerotinia sclerotiorum* causes SSR disease. The responses of the susceptible and resistant genotypes (30 and 90 days old) following inoculation as well as their respective disease progressions at 1, 2, 4, 6, 8 and 10 dpi and gene expression correlations of symptoms with levels of resistance were investigated. The goal of our study was to determine the ability of the response of resistance genes by studying the hormonal signaling pathways in response to *S*. *sclerotiorum* and to determine the relationship between the response and the lifestyle of the pathogen or type of biotic elicitor in susceptible and resistant genotypes of oilseed rape to the fungal pathogen at different times (Fig 1). The results indicate that RRCC causes less tissue damage and less severe disease symptoms compared to AACC (Fig 2). Soft-rotting necrosis of the pathogen occurred in AACC as early as 1 dpi (Fig 2A), while in the RRCC genotype necrosis occurred at 6 dpi (Fig 2B). The lesion sizes in AACC reached 0.23, 0.60, 1.00, 1.85, 2.45 and 3.11 cm at 1, 2, 4, 6, 8 and 10 dpi, respectively, whereas they reached 0.00, 0.00, 0.00, 0.20, 0.70 and 1.15 cm, respectively, in RRCC (Fig 2C).

**Fig 3:**
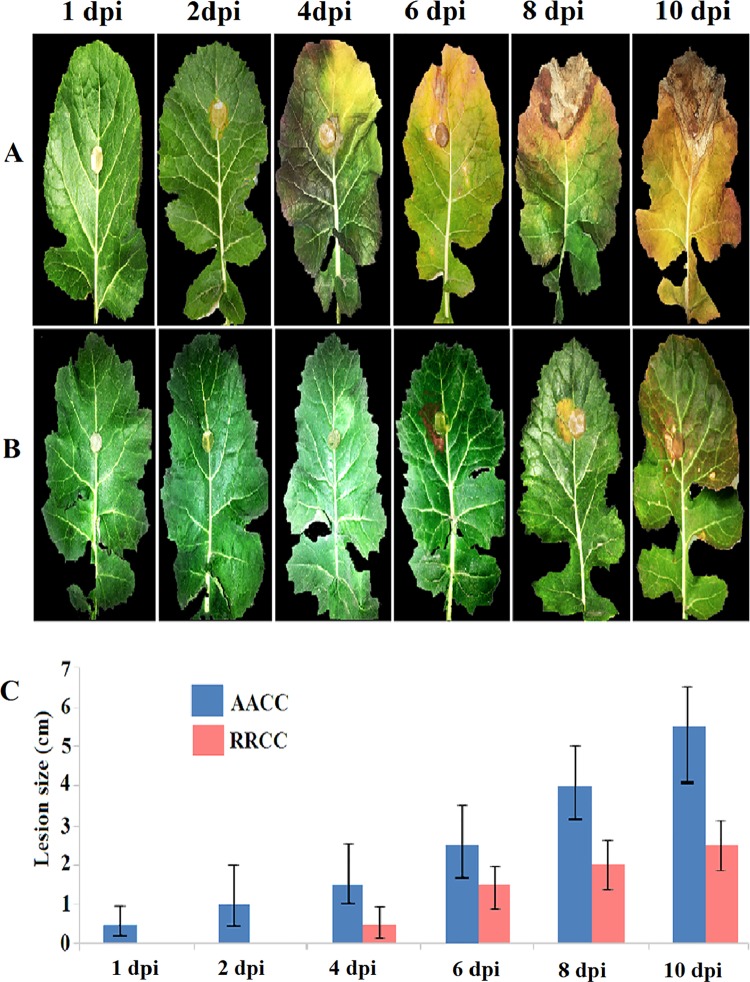
Disease progression of *S*. *sclerotiorum* infection on leaves of two genotypes: (A) *B*. *napus* AACC and (B) *R*. *alboglabra* RRCC. The leaves of both genotypes (90 days of growth) were infected with 1-cm^2^ mycelial agar discs of *S*. *sclerotiorum*. (C) Lesion size was measured 1, 2, 4, 6, 8 and 10 dpi.

The experiment also was conducted on 90-day-old material. RRCC developed less severe disease symptoms and less tissue damage than did AACC (Fig 3). Soft-rotting necrosis in AACC appeared as early as 1 dpi (Fig 3A), while necrosis occurred at 6 dpi in RRCC (Fig 3B). The lesion sizes in AACC reached 0.45, 1.15, 2.10, 2.35, 4.50 and 5.60 cm at 1, 2, 4, 6, 8 and 10 dpi, respectively, whereas they reached 0.00, 0.00, 0.50, 1.35, 1.70 and 2.85 cm, respectively, in RRCC (Fig 3C).

**Fig 4:**
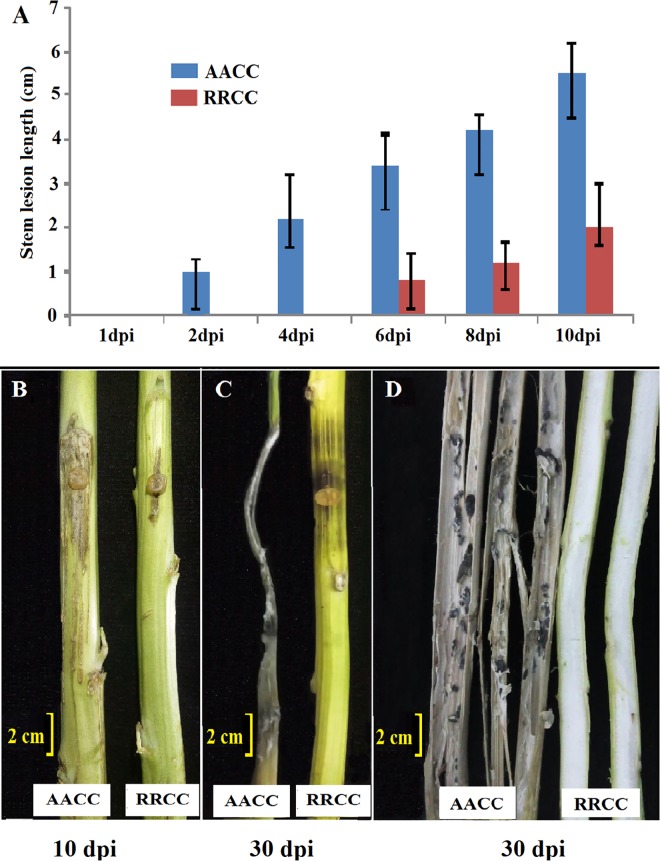
Symptoms on stems at (A) 1, 2, 4, 6, 8, 10 and 30 dpi; (B) 10 dpi; (C) 30 dpi, inside of stems; and (D) 30 dpi in partially resistant checks of *B*. *napus* AACC and *R*. *alboglabra* RRCC.

The stem resistance performance of both genotypes was assayed at the mature stage (90 days old). There was a significant difference in lesion length on the stems from 1 to 10 dpi between the two genotypes (Fig 4A). The lesion extended farther into most parts of the stem of AACC and reached 5.5 cm at 10 dpi, leading to death at 30 dpi, while the lesion extension was restrained to about 2.2 cm at 10 dpi, reaching 4.5 cm at 30 dpi on the stems of RRCC (Fig 4B and 4C). Fig 4C shows a comparison of the inside of the stems for both genotypes at 30 dpi. The sclerotia of the pathogen appeared in infected stems of *B*. *napus* but did not appear in stems of *R*. *alboglabra*.

### Effect of TH12 and CF on development of symptoms *S*. *sclerotiorum* infection

**Fig 5:**
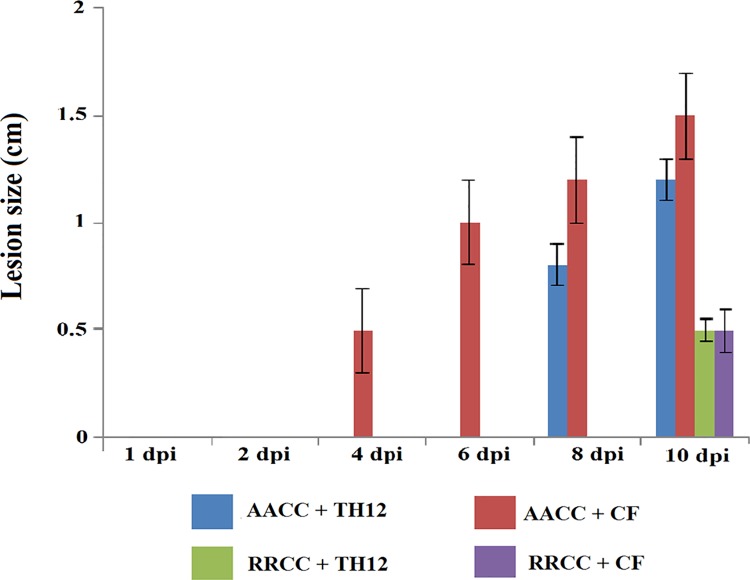
Lesion size was measured at 1, 2, 4, 6, 8 and 10 dpi of 10 mL of TH12 (1.5 ×10^7^ CFU/mL) and its CF for disease progression of *S*. *sclerotiorum* infection on leaves (30 days of growth) of two genotypes: *B*. *napus* AACC and *R*. *alboglabra* RRCC. Leaves of plants were inoculated with 1-cm^2^ mycelial agar discs of *S*. *sclerotiorum* 1 day after treatment with TH12 or CF.

The TH12 and CF biotic elicitors led to significant reduction in the lesion size of the pathogen in treated AACC and RRCC compared to un-treated controls. Soft-rotting necrosis occurred in AACC at 4 dpi (Fig 5), whereas necrosis occurred at 8 and 10 dpi in RRCC. Lesion sizes of pathogens at 1, 2, 4, 6, 8 and 10 dpi in AACC treated with TH12 reached 0.00, 0.00, 0.00, 0.50, 0.93 and 1.12 cm, respectively, while lesion sizes reached 0.00, 0.00, 0.30, 0.65, 1.00 and 1.26 cm, respectively, after being treated with CF. In RRCC treated with TH12, the lesion sizes reached 0.00, 0.00, 0.00, 0.00, 0.12 and 0.36 cm and, in those treated with CF, 0.00, 0.00, 0.00, 0.00, 0.16 and 0.48 cm at 1, 2, 4, 6, 8 and 10 dpi, respectively (Fig 5).

### Transcript levels of resistance genes in AACC and RRCC infected by *S*. *sclerotiorum*

qRT-PCR was used to detect the JA/ET and SA pathways of defense-related genes in both AACC and RRCC genotypes that were infected or not infected by *S*. *sclerotiorum* at different time periods of 1, 2, 4, 6, 8, and 10 dpi.

**Fig 6:**
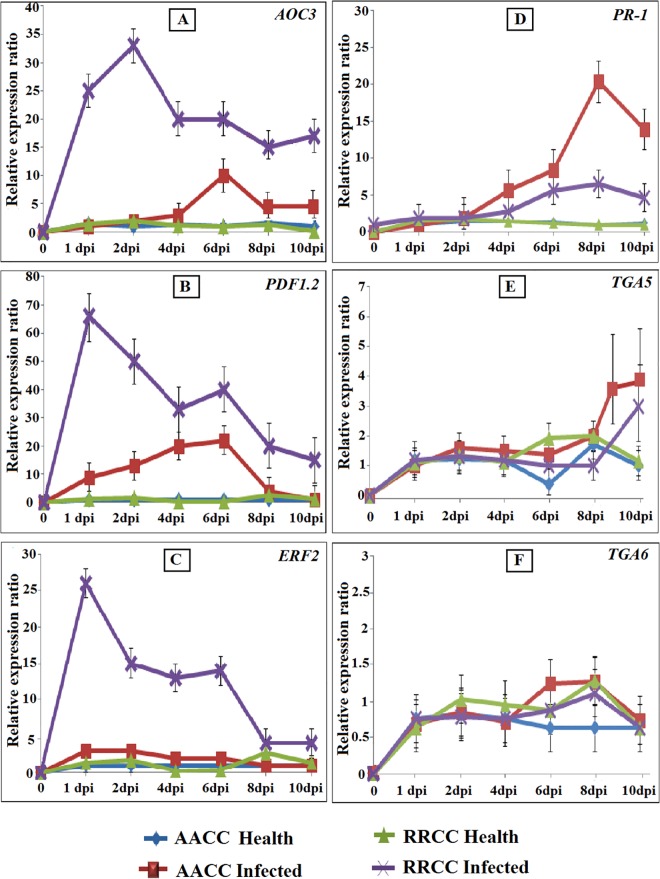
Relative gene expression in leaves (30 days of growth) of both AACC and RRCC genotypes infected with 1-cm^2^ mycelial agar discs of *S*. *sclerotiorum*. Five leaves from each pot of infected and non-infected plants were sampled 1, 2, 4, 6, 8 and 10 dpi. JA/ET-responsive genes (*AOC3*, *PDF1*.*2* and *ERF2*) and SA-inducible genes (*PR-1*, *TGA5* and *TGA6*) were analyzed with qRT-PCR and compared with *GAPDH* expression levels.

In *S*. *sclerotiorum*-infected leaves of AACC and RRCC, the expression levels of the defense-related genes, such as *AOC3*, *PDF1*.*2* and *ERF2*, were up-regulated early, as was the *PR-1* gene; however, the expression levels of *TGA5* and *TGA6* were down-regulated (Fig 6).

The expression of *AOC3* increased and peaked at 6 dpi in infected leaves of AACC and at 2 dpi in infected leaves of RRCC, increases of 10.32 and 33.22 fold, respectively (Fig 6A). Significant differences in expression levels of the *PDF1*.*2* gene occurred between the leaves infected and not infected for both genotypes. In AACC, *PDF1*.*2* increased by 22.14 fold at 6 dpi, and, in infected RRCC, it increased by 56.19 fold at 1 dpi (Fig 6B).

Expression of the *ERF2* gene, an important gene involved in the ET pathway, increased at 1 dpi by 2.63 and 25.49 fold in infected leaves of AACC and RRCC genotypes, respectively (Fig 6C). *PR-1* gene expression levels were up-regulated at 8 dpi in infected leaves of AACC and RRCC by 21.91 and 5.66 fold, respectively (Fig 6D).

*TGA5* and *TGA6* expression levels remained very low in healthy and infected leaves in both genotypes compared with expression of *AOC3*, *PDF1*.*2* and *ERF2* genes (Fig 6E and 6F).

### Gene expression levels of healthy AACC and RRCC plants treated with TH12 and CF

Plants respond actively to different elicitor treatments by deploying a range of defense-response mechanisms that eventually result in induced resistance in plants.

Expression analyses by qRT-PCR of the six genes were conducted at 1, 2, 4, 6, 8, and 10 dpi to validate whether the biotic elicitors TH12 and CF induced resistance in AACC and RRCC genotypes. The expression data was then compared between root systems of AACC and RRCC treated or not treated with biotic elicitors. The results revealed that three of the six genes (*AOC3*, *PDF1*.*2* and *ERF2*) showed the highest expression and strongest effects at all-time points among the treated seedlings.

**Fig 7:**
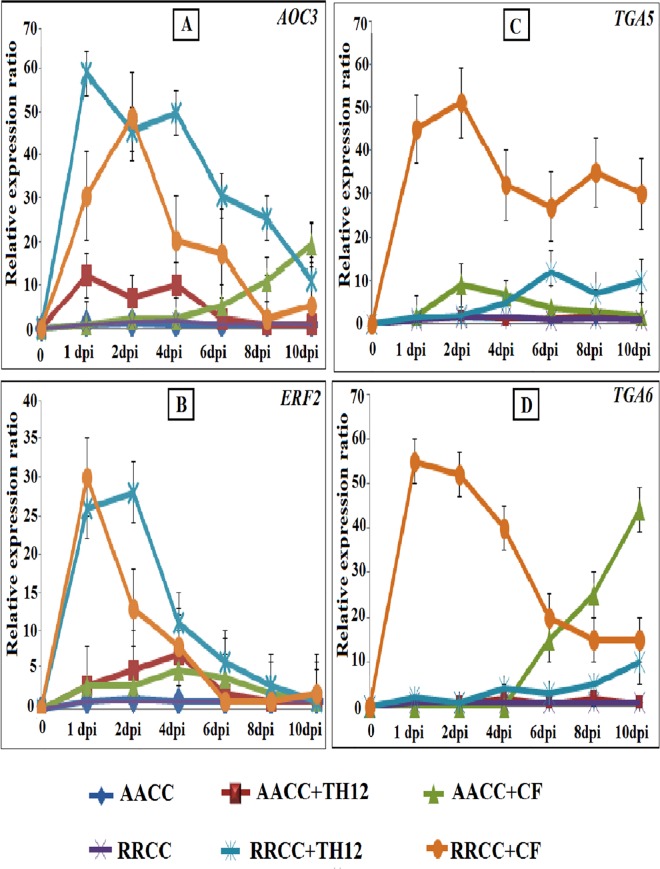
Relative gene expression in leaves (30 days of growth) of both AACC and RRCC genotypes treated or not treated with 10 mL of TH12 and its CF. Five leaves from each pot of treated and un-treated plants were sampled at 1, 2, 4, 6, 8 and 10 dpi. JA/ET-responsive genes (*AOC3*, *PDF1*.*2* and *ERF2*) and SA-inducible genes (*PR-1*, *TGA5* and *TGA6*) were analyzed with qRT-PCR and compared with *GAPDH* expression levels.

*AOC3* expression was significantly greater at 1 dpi in AACC and RRCC treated with TH12 compared to un-treated plants, reaching 11.10 and 37.01 fold, respectively. In addition, expression at 10 and 2 dpi was up-regulated in both genotypes treated with CF, reaching 17.12- and 40.89-fold higher, respectively, (Fig 7A).

The expression pattern indicated for *PDF1*.*2* in AACC and RRCC was up-regulated in response to TH12 treatment by 6 and 1 dpi and increased by 25.03 and 81.69 fold, respectively. The *PDF1*.*2* gene expression levels were significantly higher in seedlings treated with CF compared to un-treated seedlings; the present experiment verified our previous results (S1 Fig).

The *ERF2* expression pattern also was up-regulated in both genotypes, reaching 5.36- and 3.94-fold higher at 4 dpi in AACC treated with TH12 and CF, respectively, and reaching 27.36- and 26.14-fold higher in RRCC treated with TH12 at 2 dpi and CF at 1 dpi, respectively (Fig 7B).

The involvement of the SA signaling pathway is further supported by marker genes *PR-1*, *TGA5* and *TGA6*. The expression levels of *PR-1* after treatment with CF increased in AACC at 2 dpi and RRCC at 4 dpi by 17.91 and 21.92 fold, respectively, which was similar to our previous results (S1 Fig) [16]. The expression levels of *TGA5* increased in AACC and RRCC treated with CF by 7.27 and 42.89 fold, respectively, at 2 dpi, and reached 11.25 fold at 6 dpi in RRCC treated with TH12 (Fig 7C). The expression levels of the *TGA6* gene increased by 55.74 fold at 1 dpi in RRCC and by 42.53 fold at 10 dpi in AACC treated with TH12 (Fig 7D).

These results indicate the role of biotic elicitors in activating signaling in seedlings of both genotypes by measuring expression levels of resistance genes that had previously been used as markers of resistance pathways and, therefore, indicate their ability to induce systemic resistance in plants.

### Effects of TH12 and CF on resistance gene expression levels in AACC and RRCC infected by *S*. *sclerotiorum*

In order to examine the expression of resistance genes related to JA/ET and SA pathways in response to TH12 and CF treatment and pathogen infection, 30-day-old susceptible and resistant oilseed rape seedling root systems were drench-treated with the biotic elicitors, and after 1 day the leaves were inoculated with mycelia of *S*. *sclerotiorum*. Leaf samples were taken daily at 1, 2, 4, 6, 8 and 10 dpi for subsequent qRT-PCR assessment.

**Fig 8:**
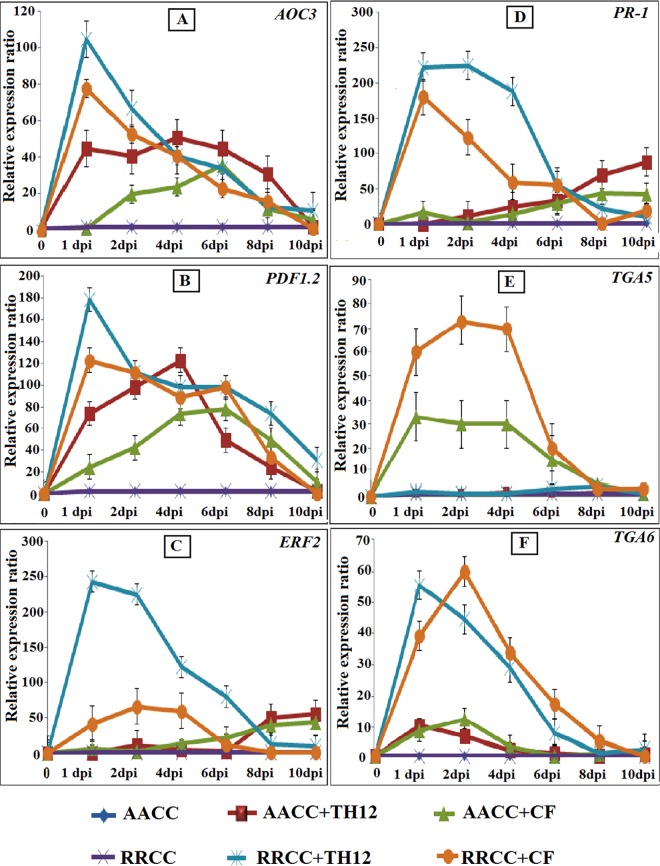
Relative gene expression in leaves (30 days of growth) of both AACC and RRCC genotypes treated or not treated with 10 mL of TH12 and its CF separately by soil drenching; after 1 day the leaves were inoculated with 1-cm^2^ mycelial agar discs of *S*. *sclerotiorum*. Five leaves from each pot of treated and un-treated plants were sampled at 1, 2, 4, 6, 8 and 10 dpi. JA/ET-responsive genes (*AOC3*, *PDF1*.*2* and *ERF2*) and SA-inducible genes (*PR-1*, *TGA5* and *TGA6*) were analyzed with qRT-PCR and compared with *GAPDH* expression levels.

Upon *S*. *sclerotiorum* infection, expression levels of resistance genes were induced dramatically in seedlings that were treated in comparison with those not treated after 1 day, when the lesion size area began to decrease on the leaves treated with biotic elicitors (Fig 5), whereas the *AOC3* gene expression levels increased by 95.15 and 72.52 fold at 1 dpi in RRCC treated with TH12 and CF suspension, respectively, and the same levels increased by 43.80 and 31.71 fold at 4 and 6 dpi, respectively, in AACC (Fig 8A). The levels of *PDF1*.*2* in RRCC increased by 173.70 fold after being treated with TH12 and reached 115.88-fold higher after being treated with CF at 1 dpi. In AACC treated with TH12, the levels of *PDF1*.*2* increased by 122.90 fold at 4 dpi and reached 67.21-fold higher at 6 dpi after treatment with CF (Fig 8B). The levels of the RRCC *ERF2* gene treated with TH12 were up-regulated by 247.10 fold at 1 dpi, and the levels reached 66.18 fold at 2 dpi when treated with CF compared to un-treated plants (Fig 8C). In AACC treated with TH12 and CF, *ERF2* increased by 54.12 and 41.49 fold at 10 dpi, respectively (Fig 8C).

In RRCC seedlings treated with TH12 and CF, *PR-1* gene expression levels increased by 229.17 and 177.63 fold at 1 dpi, respectively, whereas in AACC *PR-1* expression levels increased by 88.47 and 39.61 fold at 10 dpi, respectively (Fig 8D).

*TGA5* expression increased 73.60 and 30.37 fold after treatment of RRCC and AACC with CF, respectively (Fig 8E). Basal *TGA5* expression levels did not vary much between RRCC and AACC seedlings.

For the *TGA6* gene, expression levels in RRCC reached 47.67-fold after induction with TH12 and 41.08-fold after induction with CF higher at 1 dpi and 2 dpi, respectively, while in AACC they reached 10.73 and 12.26-fold higher, respectively (Fig 8F).

## Discussion

*Sclerotinia sclerotiorum* is a necrotrophic, soil-borne, non-specific pathogen that can attack more than 400 hosts, including many economically important crops. Necrotrophic pathogens overcome the defenses of various hosts by using a relatively straightforward strategy employing cell wall-degrading enzymes and killing the host cell by toxins, using the cells as their source of nutrients [33]. This study laid emphasis on the resistance signaling pathways in genotype of oilseed rape–a susceptible *B*. *napus* AACC and a resistant genotype *R*. *alboglabra* RRCC–involving the *S*. *sclerotiorum* pathogen and analyzed the effectiveness of *T*. *harzianum* TH12 and its cell-free CF root treatment in the enhancement of resistance against this pathogen.

The virulence measurements showed the necrotic lesions of *S*. *sclerotiorum* increased soon after inoculation on the AACC leaf surface but did not increase rapidly in RRCC leaves. The interplay between *S*. *sclerotiorum* and the genotypes of the resistant RRCC or susceptible AACC were shown by 1 and 2 dpi (Fig 2). The differences in size of necrotic lesions on AACC and RRCC leaves led us to hypothesize that the hormone signaling pathways are different between the two genotypes.

The present study showed that *B*. *napus* used in this experiment is susceptible to infection but that *R*. *alboglabra* is resistant to infection by *S*. *sclerotiorum*. Previous studies also have shown that *B*. *napus* is susceptible to *S*. *sclerotiorum* [2, 34–36], whereas various resistant genotypes of oilseed have been observed, including *R*. *alboglabra* to *S*. *sclerotiorum* [2], *B*. *napus* ‘Charlton’ cultivar [34, 35] and *Brassica oleracea* [37] as well as the hexaploid *B*. *napus* (AACCCC), derived from crosses between ‘Zhongshuang 9’ and *B*. *oleracea*-related *B*. *incana* genotype ‘C01’ [36]. In *R*. *alboglabra*, fungal invasion was confined to the upper epidermis, which might be due to strong defense responses in the resistant genotypes, including post-penetration cell death or the hypersensitive reaction, as evidenced by the localized necrosis due to death of palisade mesophyll cells near the site of infection at 6 dpi [38, 39], reactive oxygen species (ROS) accumulation and callose deposition [38, 39] and by production of cell wall appositions (papillae) and other classes of compounds, including cell wall proteins, phenolics and cell wall polymers, such as 1,3-β-glucan callose [40, 41].

The phytohormones JA, ET, SA and abscisic acid (ABA) comprise a complex set of hormone signaling pathways that lead to the regulation of resistance in plants against necrotrophic pathogens [42]. More accurately, the plant resistance system is believed to rely preferentially on the JA/ET pathway-dependent defenses against necrotrophic lifestyle pathogens [25, 43]. Previous studies reported that the JA/ET pathways defenses were induced by necrotrophic fungi, whereas herbivorous insects and biotrophic pathogens lead to induction of the SA pathway [44, 45]. We used *AOC3* (as a JA pathway marker), *PDF1*.*2* (as a JA/ET pathway marker) and *ERF2* (as an ET pathway marker) as well as *PR-1*, *TGA5* and *TGA6* (as SA pathway markers) genes. Six genes were used in this study because of their ability to be involved in the resistance against pathogens and their activities after being induced by external elicitors or in the attack of pathogens, in addition to being used as markers for resistance pathways in sensitive or resistant genotypes of oilseed rape [16, 25]. The expressions of *AOC3*, *ERF2* and *PDF1*.*2* were all significantly different at 1 and 2 dpi in the leaves of RRCC infected by *S*. *sclerotiorum*, This refers to the high response of resistance genes against the patient within a short period after infection may be the cause of RRCC genotype ability to resist disease, therefore, we find in the case of AACC, expression of these genes increased at 8 and 10 dpi.

The expression levels of *PR-1* were up-regulated at 8 and 10 dpi in AACC and RRCC, respectively, this result proves that *PR-1* gene is a molecular marker for the SA signaling pathway and the SAR response against biotrophic pathogens because the response came too late in both genotypes infected by necrotrophic pathogen, which is in accordance with recent studies that showed the involvement of JA, ET and SA signaling in infected leaves of two varieties of *B*. *napus* that were sensitive or resistant to *S*. *sclerotiorum* [46, 47]. Our results differ with those studies regarding time, as our results demonstrated that SA signaling was activated later, at 6–10 dpi, while those studies confirmed that SA signaling was activated earlier, at 12 h [46] or 48 h post infection [47]. SA-regulated *PR-1*, *TGA5* and *TGA6* expression resulted in enhanced plant susceptibility of *B*. *napus* to *S*. *sclerotiorum* and reduced expression of JA/ET-regulated *AOC3*, *PDF1*.*2* and *ERF2*, indicating that SA and JA/ET signaling pathways act antagonistically [39]. Blocking SA accumulation can enhance JA/ET accumulation and signaling in sclerotinia-infected RRCC, resulting in elevated resistance to necrotrophic pathogens. Likewise, blocking JA/ET accumulation can promote SA-regulated genes in the *R*. *alboglabra* genotype after infection by the obligate parasite *E*. *cruciferarum* [16].

*PR-1*, *TGA5* and *TGA6* are important for SA accumulation and have essential functions in the positive regulation of systemic acquired resistance SAR in against various biotic and abiotic stresses [48]. In addition, inoculation of plants with the necrotrophic fungi led to a marked increase in production of ethylene or jasmonic acid pathways for the induction of resistance genes. *PDF1*.*2*, *AOC3* and ERF family, including *ERF2* are induced resistance response to pathogen infection is activated synergistically by ethylene and jasmonic acid (JA) [23].

This study has shown that a strong response of ET signaling (*ERF2* and *PDF1*.*2*) was induced in RRCC during infection by *S*. *sclerotiorum* at 1 dpi, followed by a subsequent induction of JA signaling (*AOC3*), which refers to involved JA and ET pathways in the resistance against the necrotrophic fungi. Earlier studies that used qRT-PCR analysis of an oilseed rape crop infected by *S*. *sclerotiorum* showed an increase in the expression levels of genes related to the JA/ET signaling pathways [25, 49].

Our previous studies demonstrated that *T*. *harzianum* TH12 and its CF were effective biotic elicitors, with high mycoparasitic activity, and have the ability to stimulate resistance and to reduce the disease index and degree of infection in AACC and RRCC when used in soil drenching against *S*. *sclerotiorum* [2] or in the spraying of leaves and stems against the obligate parasite *E*. *cruciferarum* [9].

*Trichoderma* spp. directly impacts plant production of phytohormone-like molecules, secondary metabolites and volatile organic compounds or alters plant phytohormone homeostasis [50, 51]. *Trichoderma* spp. can increase plant development and protection through release of some elicitors that may contribute to signals being transmitted within the plants, such as JA, SA and ROS [52]. In this study, we examined the presence of *S*. *sclerotiorum* in the leaves and *Trichoderma* isolate TH12 and CF in the roots, and the pathogen remained physically separated from the biotic elicitors. *Trichoderma harzianum* TH12 and its CF reduced the necrotrophic lesion size in both treated genotypes, and the results provide compelling evidence that TH12 and its CF can induce systemic resistance. These results showed signal transfer of resistance from the rhizosphere to the phyllosphere that renders distal tissue parts resistant to *S*. *sclerotiorum*, and the results indicate that the *R*. *alboglabra* genotype treated with biotic elicitors contains a high systemic resistance and a long-distance systemic resistance-inducing signal synthesized in the treated roots compared to AACC. The results showed increased protection from TH12 and its CF on plants infected or not infected in both genotypes, which was most likely related to mechanisms of plant defense induced by the release of elicitors from hyphae, polysaccharides or secondary metabolites of *Trichoderma* spp. Other studies have shown that different *T*. *harzianum* strains can induce systemic resistance against necrotrophic pathogens in different crops [15, 53–55].

Comparative gene expression levels showed that the treatment of AACC and RRCC genotypes with TH12 and its CF resulted in a significant change in the expression levels in these plants when infected with *S*. *sclerotiorum* compared to non-infected plants. Specifically, RRCC seedlings treated with TH12 and its CF responded to the inoculation with stronger and faster induction of genes related to the JA/ET pathways (i.e., *AOC3*, *PDF1*.*2* and *ERF2*). Defense-related genes involved in the SA pathways, including *PR-1*, *TGA5* and *TGA6*, also were differentially expressed in RRCC and AACC treated with CF. We found a significant increase in gene expression levels of *AOC3*, *PDF1*.*2* and *ERF2* at 1 and 2 dpi in RRCC, suggesting that both genotypes quickly respond to TH12 and its CF, but the levels of *PR-1*, *TGA5* and *TGA6* genes did not increase in either genotype treated with TH12 compared with those treated with CF. These results indicate that TH12 induces resistance through the JA/ET pathways, as seen by the boosted expression of the JA-regulated *PDF1*.*2* and *PR-3* marker genes. The current results are consistent with those of several studies showing that *T*. *harzianum* induces systemic resistance against pathogens in plants through a boosted JA-dependent defense response [36, 56].

Our results indicate that increased resistance gene expression levels related to the ET signaling pathway in the oilseed rape crop or treatment with TH12 and its CF can reduce the symptoms of necrotic lesions of *S*. *sclerotiorum*. Previous results have indicated that ET signaling seems to reduce symptom development of necrotrophic *S*. *sclerotiorum* in oilseed [16] and in *Arabidopsis thaliana* [57]. Interestingly, levels of *PR-1* and *TGA5* increased early in RRCC infected with *S*. *sclerotiorum* and treated with TH12, but they decreased in healthy RRCC treated with TH12. These results indicate that genes related to the SA signaling pathway were stimulated as a result of the necrotrophic pathogen infection after treatment with the biotic elicitor together, which shows that *S*. *sclerotiorum* is involved in SAR via the SA pathway. It is possible that during ISR, defensive genes have accumulated so that the levels of these genes at the moment of *S*. *sclerotiorum* inoculation are already higher in TH12-treated plants than in non-inoculated plants. This would rule out the need for a further strong or fast induction of the defense response at the gene expression level and, hence, would lead to the observed reduction of the ISR defense response [58]. Moreover, the expression levels of SA, JA and ET pathways in seedlings treated with CF and in seedlings infected or not infected with pathogens were up-regulated. These results suggest that CF plays a role in SAR in the resistance or susceptibility of oilseed rape genotypes by stimulating SA signaling pathways against *S*. *sclerotiorum*. Some reports have shown a role of cell-free filtrates of *Trichoderma* spp. in induced resistance in *A*. *thaliana* and that the filtrates induce both the JA/ET and SA signaling pathways against pathogens [59, 60]. RRCC genotype was protected early than AACC after application of the CF, gene expression experiments revealed that CF treatment induced the systemic expression of both genes related with JA/ET and SA pathways. In conclusion, our data suggest that CF induces resistance in RRCC and AACC in a manner where JA/ET and SA pathways may play a role in defence signalling.

## Conclusion

Our results show that the JA/ET signaling pathway was activated in a resistant genotype of oilseed rape at the early stage of infection with a necrotrophic pathogen, that the pathway increased in resistant and susceptible genotypes after treatment with biotic elicitors, such as TH12, but that the SA signaling pathway was activated at an early stage after treatment with CF biotic elicitors. We have demonstrated that these pathways depend on the type of elicitor; that they are used to establish resistance in systemic oilseed rape tissues to *S*. *sclerotiorum*; and that SA, JA and ET pathways are involved in conferring SAR or ISR responses. Further studies of pathways related to systemic and local resistance marker genes will help to refine the RRCC genotype.

## Supporting Information

S1 Fig Expression of defense-related genes of AACC and RCC genotypes of inoculated with TH12 and its cell-free culture filtrate (CF) separately. Leaves were collected 1, 2, 4, 6, 8 and 10 days post-inoculation. Total RNA was extracted, and cDNA was synthesized. Expression levels of the *PR-1*, *PR-2*, *PDF1*.*2* (glucanase; BGL2), *PR-3* (basic chitinase), *CHI620* and *CHI570* (chitinase) genes were monitored by RT q- PCR. The expression levels of genes were compared with the expression level of *GAPDH* [16].
(TIF)
