# Supplementary figures and images for: Detecting the Hormonal Pathways in Oilseed Rape behind Induced Systemic Resistance by Trichoderma harzianum TH12 to Sclerotinia sclerotiorum
Source: PLoS One. 2017 Jan 3;12(1):e0168850. doi: 10.1371/journal.pone.0168850 (PMC5207704; doi:10.1371/journal.pone.0168850)

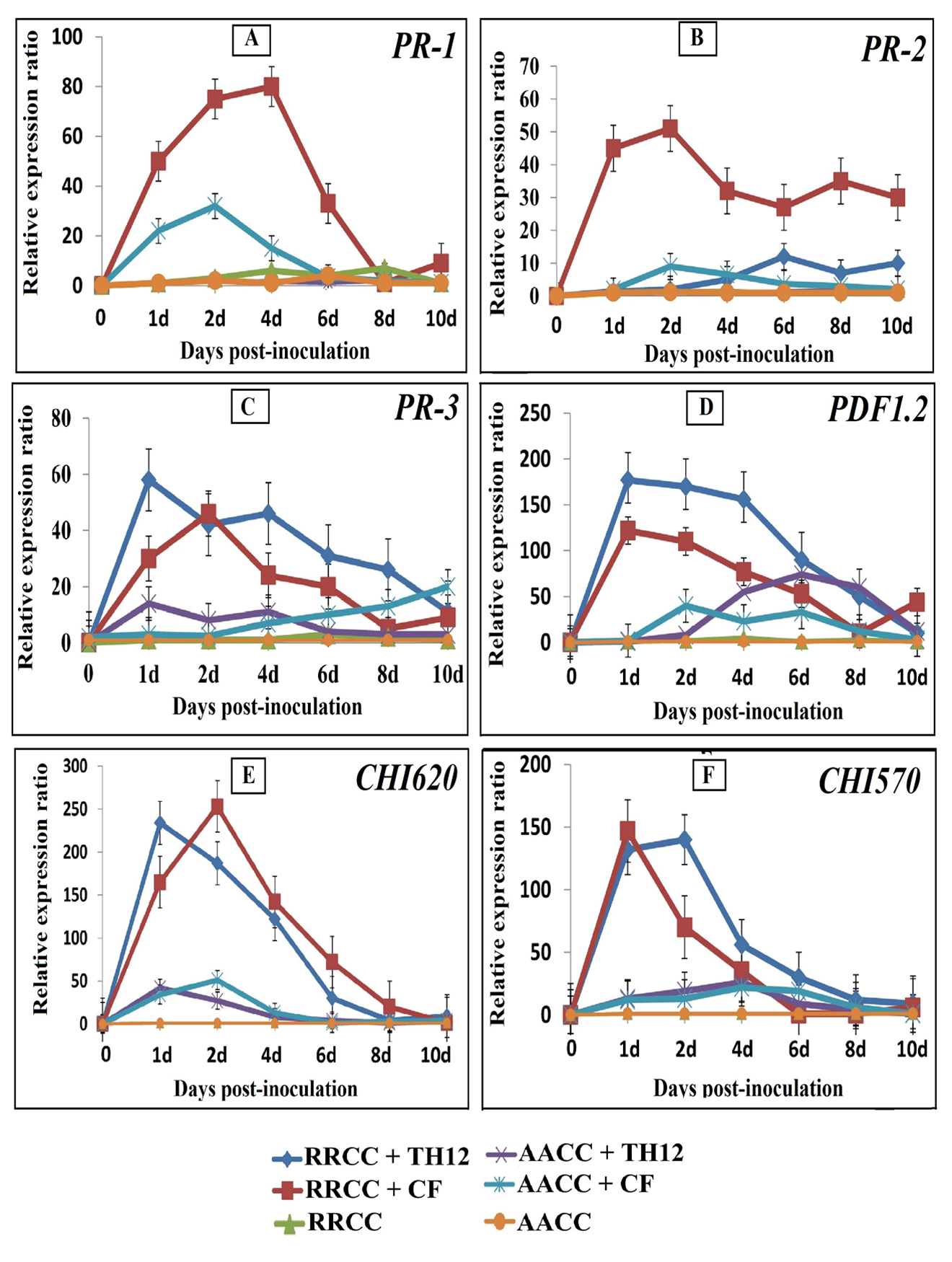

Supplement: S1 Fig — Leaves were collected 1, 2, 4, 6, 8 and 10 days post-inoculation. Total RNA was extracted, and cDNA was synthesized. Expression levels of the PR-1, PR-2, PDF1.2 (glucanase; BGL2), PR-3 (basic chitinase), CHI620 and CHI570 (chitinase) genes were monitored by RT q- PCR. The expression levels of genes were compared with the expression level of GAPDH [16]. (TIF) [file pone.0168850.s001.tif]
